# Biomarkers of mammographic density in premenopausal women

**DOI:** 10.1186/s13058-021-01454-3

**Published:** 2021-07-23

**Authors:** Mathilde His, Martin Lajous, Liliana Gómez-Flores-Ramos, Adriana Monge, Laure Dossus, Vivian Viallon, Audrey Gicquiau, Carine Biessy, Marc J. Gunter, Sabina Rinaldi

**Affiliations:** 1 International Agency for Research on Cancer (IARC/WHO), Nutrition and Metabolism Branch, CEDEX 08, 69372 Lyon, France; 2 Center for Research on Population Health, National Institute of Public Health, 62100 Cuernavaca, México; 3 Department of Global Health and Population, Harvard T.H. Chan School of Public Health, Boston, MA 02115 USA; 4 Cátedras-CONACYT, Mexico City, Mexico

**Keywords:** Mammographic density, Premenopausal, Mexican women, Targeted metabolomics

## Abstract

**Background:**

While mammographic density is one of the strongest risk factors for breast cancer, little is known about its determinants, especially in young women. We applied targeted metabolomics to identify circulating metabolites specifically associated with mammographic density in premenopausal women. Then, we aimed to identify potential correlates of these biomarkers to guide future research on potential modifiable determinants of mammographic density.

**Methods:**

A total of 132 metabolites (acylcarnitines, amino acids, biogenic amines, glycerophospholipids, sphingolipids, hexose) were measured by tandem liquid chromatography/mass spectrometry in plasma samples from 573 premenopausal participants in the Mexican Teachers’ Cohort. Associations between metabolites and percent mammographic density were assessed using linear regression models, adjusting for breast cancer risk factors and accounting for multiple tests. Mean concentrations of metabolites associated with percent mammographic density were estimated across levels of several lifestyle and metabolic factors.

**Results:**

Sphingomyelin (SM) C16:1 and phosphatidylcholine (PC) ae C30:2 were inversely associated with percent mammographic density after correction for multiple tests. Linear trends with percent mammographic density were observed for SM C16:1 only in women with body mass index (BMI) below the median (27.4) and for PC ae C30:2 in women with a BMI over the median. SM C16:1 and PC ae C30:2 concentrations were positively associated with cholesterol (total and HDL) and inversely associated with number of metabolic syndrome components.

**Conclusions:**

We identified new biomarkers associated with mammographic density in young women. The association of these biomarkers with mammographic density and metabolic parameters may provide new perspectives to support future preventive actions for breast cancer.

**Supplementary Information:**

The online version contains supplementary material available at 10.1186/s13058-021-01454-3.

## Background

Mammographic density reflects the amount of stromal and epithelial tissue in the breast, i.e., radiopaque components, in contrast with fat tissue [1]. Mammographic density is approximately 60% heritable [2], but is also associated with non-heritable factors such as age and menopausal status [3], parity and number of children [4], use of hormone replacement therapy [5], obesity [6, 7], and alcohol intake [8, 9]. Improving knowledge on factors that influence mammographic density is crucial, since mammographic density is among the most important risk factors for breast cancer. Women with a high mammographic density have an increased risk for breast cancer, that is estimated to be 4.6 times higher for mammographic density ≥ 75% compared with < 5% [10]. The direct association between high mammographic density and risk of breast cancer has been observed both in post- and premenopausal women [11]. However, since premenopausal women are not targeted in existing mammography screening programs, it is not possible to identify premenopausal women with a high mammographic density, at increased risk for breast cancer. The identification of specific biomarkers for high mammographic density may represent a valuable alternative to mammography to identify women at higher breast cancer risk.

While several potential biomarkers of mammographic density have been investigated in premenopausal women, such as growth factors [12–14], leptin [15], or sex steroids [12, 16], results remain mostly inconsistent. Metabolomics is a technique able to detect subtle changes in metabolism, which has been useful in identifying new biomarkers associated with breast cancer risk, pointing to new etiological hypotheses [17–21]. Applying this technique to mammographic density may provide new insights into the understanding of the determinants of mammographic density and its association with breast cancer risk.

In this work, we used a targeted metabolomics approach to identify potential metabolites specifically associated with mammographic density. To do so, 132 metabolites were measured in 573 plasma samples from a cross-sectional study of premenopausal women enrolled in the Mexican Teacher’s Cohort with available mammographic density measures. In addition, to provide a better understanding of the metabolites associated with mammographic density, we investigated potential correlates of their plasma concentrations.

## Methods

### The Mexican Teachers’ Cohort (MTC)

The MTC has been described in detail elsewhere [22]. In brief, this cohort study started with enrollment of 27,979 female teachers from the Mexican states of Jalisco and Veracruz in 2006, before the recruitment was extended to other states of Mexico in 2008 to reach a total of 115,314 female participants. With the aim to characterize risk factors related to cancer and other chronic diseases, women were administered lifestyle and dietary questionnaires. In 2007, a subsample of 2,045 women from the regions of Jalisco and Veracruz participated in a clinical examination that included an interview, anthropometric measurements by trained personnel, a mammogram, and biological samples collection. All participants gave informed consent for future use of biological specimens and questionnaire data. The International Agency for Research on Cancer (IARC) Ethics Committee as well as the Research Ethics Committee at the National Institute of Public Health in Cuernavaca, Mexico, approved the current project.

### Blood collection and storage

Trained nurses collected fasting blood samples (25 mL). Plasma, serum, erythrocytes, and buffy coat were separated by centrifugation at 2500 rpm for 10 min in a refrigerated centrifuge (4 °C) and aliquoted into several cryotubes at field work site, within 30 min after blood collection. Samples were frozen and kept in liquid nitrogen at the National Institute of Public Health in Cuernavaca, Mexico, until shipment to IARC, where they were stored at − 80 °C until metabolomics analyses were run.

### Selection of the population

Out of the 2045 women who underwent clinical examination, 230 with missing information on metabolic syndrome components were excluded (based on inclusion criteria for a previous study [23]), 67 because of unknown menopausal status, and 624 who were postmenopausal at the time of their mammogram.

Menopausal status was defined as follows: premenopausal if women menstruated at least once over the 12 months prior to recruitment, postmenopausal if women had (1) no menstruation over the last 12 months prior to the clinical examination or (2) surgical menopause (reported bilateral oophorectomy or reported unknown surgery) and were over 48 years old (mean age at menopause in Mexico [24]).

Further selection was based on commonly used [25] breast density categories (< 10%, 10 to < 25%, 25 to < 50%, ≥ 50%): women from each group of breast density were randomly selected proportionally to the size of the group, among non-users of oral contraceptive at blood donation. A total of 35 women were selected for the first group, 158 for the second, 247 for the third, and 160 for the last group. Among the 600 women whose samples were analyzed for targeted metabolomics, 1 woman was excluded because she had no biological sample, 7 women were excluded because they were older than 55 but declared to be premenopausal, and 19 women for whom measured BMI was not available at the time of clinical evaluation. Our final population included 573 women.

### Mammographic density measurement

A radiology technician performed mammography in both Jalisco and Veracruz. Cranio-caudal views were taken on each breast. Analog films were digitized using an Astra 2400S (Umax, Fremont, CA). Mammograms of both states were combined, and mammographic density was measured by a single observer on the cranio-caudal view of the left breast using Mamgr, a computer-assisted program developed at the Department of Epidemiology and Population Health, London School of Hygiene and Tropical Medicine, and based on previously reported mammographic density assessment methods [26]. In a validation study, intraclass correlation coefficient between mammographic density measurements performed with the Mamgr software versus with the Cumulus program was 0.87 (*n* = 100 mammograms), while the intra-observer intraclass correlation coefficient was 0.84 (*n* = 108 duplicates mammograms) [27]. Percent mammographic density was automatically calculated as the percent of dense pixels within the breast area. Non-dense area was calculated by subtracting the dense area from the total breast area. We converted absolute dense and non-dense area values to cm^2^ according to pixel size used in digitalization.

### Metabolites measurements

All plasma samples were assayed in the laboratory of the Biomarkers Group at IARC by liquid chromatography mass spectrometry using the Absolute*IDQ* p180 kit (Biocrates Life Sciences AG, Innsbruck, Austria) and following the procedure recommended by the vendor. A QTRAP5500 mass spectrometer (AB Sciex, Framingham, MA, USA) was used to measure 143 metabolites (17 acylcarnitines, 21 amino acids, 12 biogenic amines, 78 glycerophospholipids, 14 sphingolipids and hexoses). Samples from Jalisco and Veracruz centers were analyzed in separate batches.

### Selection of metabolites

Metabolites were analyzed in samples from 599 participants. Values lower than the lower limit of quantification (LLOQ), as well as lower than batch-specific limit of detection (LOD) (for compounds measured with a semi-quantitative method: acylcarnitines, glycerophospholipids, sphingolipids), or higher than the upper limit of quantification (ULOQ), were considered out of the measurable range. Metabolites were excluded from the statistical analyses if more than 20% of observations were outside the measurable range (*n* = 11; 9 lower than LOD or LLOQ; 2 greater than ULOQ). A total of 132 metabolites (12 acylcarnitines, 21 amino acids, 7 biogenic amines, 77 glycerophospholipids, 14 sphingolipids and hexoses) were finally retained for statistical analyses. Of these 132 metabolites, 2 had values above the ULOQ (arginine (1.8%) and taurine (17.3%)) and were imputed with the ULOQ, and 9 had lower than LLOQ or LOD (≤ 9.0%) and were imputed with half the LLOQ or half the batch-specific LOD, respectively. The remaining 121 metabolites had all values included in the measurable range.

Percent of samples out of the measurable range and coefficients of variation for metabolites included in the analysis (median = 6.0%, interquartile range = 2.1%) are shown in Supplementary Table 1.

### Covariate data

Data on dietary habits were collected through a 139-item food frequency questionnaire (details previously published [28]). Information on frequency of consumption and portion size were used to calculate nutrients and energy intakes using the United States Department of Agriculture food-composition database and the Mexican National Health and Nutrition Survey database. Three dietary patterns were identified by principal component analysis (“Fruits and Vegetables,” “Western,” “Modern Mexican”) [28]. Intakes and frequency were also used to estimate the Healthy Eating Index (HEI) 2015 total score [29].

Insulin-like growth factor 1 (IGF-1), IGF binding protein 3 (IGFBP-3), C-peptide, C reactive protein (CRP), leptin, and adiponectin analyses were performed in the laboratory of the Biomarkers Group at IARC [30]. Serum IGF-I, IGFBP3, and C-peptide concentrations were measured by immunoradiometric assays by Beckmann Coulter (Marseille, France) [13]. Leptin was measured by a radioimmunoassay from Linco (Millipore, Billerica, MA, USA), while adiponectin and CRP were measured using an enzyme-linked immunoassay by R&D (R&D Systems, Europe, Lille, France) [15].

Triglycerides, total and HDL cholesterol, and glucose were measured on fasting plasma blood samples at the Endocrinology and Metabolism Laboratory at the National Institute of Nutrition and Medical Sciences using standard assays. Glucose was measured via the automatized glucose oxidase method; triglycerides and HDLs were measured using enzymatic hydrolysis in an automatic analyzer with a tungsten lamp (Prestige 24i, Tokyo Boeki Medical System LTD). Number of metabolic syndrome components was defined according to the harmonized definition [31] (waist circumference ≥ 88 cm, triglyceride levels ≥ 150 mg/dL, HDL cholesterol levels < 50 mg/dL, systolic blood pressure > 130 mmHg or diastolic blood pressure > 85 mmHg, and glucose levels ≥ 100 mg/dL) (details previously published [23]).

### Statistical analyses

Descriptive analyses were performed for selected characteristics of the population using mean and standard deviation (continuous variables) or frequency (categorical variables). Partial Pearson’s correlation coefficients, adjusted for age (where appropriate), state, and batch were computed for metabolites, measures of mammographic density, age, and BMI. Percent mammographic density was the primary outcome of this analysis, while dense area and non-dense area were examined as secondary outcomes after log-transformation to better approximate normality and homoscedasticity of the residuals. To account for analytical batch, residuals of log-transformed and standardized metabolites concentrations were obtained from linear models with random effect for analytical batches. These residuals were used as dependent variables in multiple linear regression testing associations with the different outcomes.

All models were adjusted for a priori selected breast cancer risk factors that included: age (continuous), BMI (continuous), age at menarche (< 12, 12, 13, ≥ 14 years, missing), family history of cancer (yes, no), history of benign breast disease (yes, no), use of oral contraceptive (ever, never), number of full-term pregnancies (0, 1, 2, 3, ≥ 4, missing), age at first full-term pregnancy (nulliparous, < 20, 20–25, 25–30, ≥ 30, missing), breastfeeding (nulliparous, no breastfeeding, < 6 months, 6–12 months, 12–24 months, ≥ 24 months, missing), alcohol intake (0, 0.1 drinks/day, 0.1–0.2 drinks/day, ≥ 0.2 drinks/day, missing), smoking status (never, past, current, missing), socioeconomic status (low, medium, high, missing), and physical activity (continuous). A missing category was created for all variables, except for physical activity where the only missing value was imputed to the median. Multiple tests were addressed using permutation *minP*-adjustment of *P* values to account for the dependencies between tests [32].

For metabolites associated with percent mammographic density after correction for multiple testing, adjusted means of percent mammographic density were estimated by quartile of metabolite. For test of linear trend, participants were assigned the median value of exposure in each quartile and we modeled the corresponding variable as a continuous term. Analyses were further stratified by BMI (</> 27.4 kg/m^2^ (median)) and interaction with the dichotomized variable was tested for each metabolite by including an interaction term in the model. Adjusted means were examined by quartiles of metabolite in each group, and BMI (continuous) was included as an adjustment variable in each model.

To examine the robustness of the observed associations, additional exploratory analyses were conducted using a bootstrapped least absolute shrinkage and selection operator (LASSO) regression approach [33, 34]: metabolic signatures of the percent mammographic density were obtained via simple cross-validated LASSO, which efficiently selects the most predictive variables in high-dimensional sets of potential predictors. This approach was then applied to 200 bootstrap samples to determine which metabolites were most frequently included in the signature.

To provide a better understanding of the metabolites associated with percent mammographic density, a variety of lifestyle, dietary, anthropometric, and metabolic factors already available in the study population were investigated in separate models in relation to plasma concentration of retained metabolites. Adjusted mean concentrations of metabolites of interest (residuals on analytical batch) were estimated across categories of each variable after excluding participants with missing values, adjusting for age and state. All variables previously listed as covariates in the main analyses were examined using similar categories or tertiles for variables initially included as continuous. In addition to these variables, we investigated waist circumference (tertiles), hip circumference (tertiles), high blood pressure (yes, no), circulating leptin, adiponectin, leptin/adiponectin ratio, IGF-1, IGFBP-3, C-peptide, CRP (tertiles of log-transformed concentration regressed on respective analytical batches), total cholesterol (tertiles), HDL cholesterol (tertiles), total cholesterol/HDL cholesterol ratio (tertiles), triglycerides (tertiles), glucose (tertiles), and number of criteria for determination of metabolic syndrome. The following nutritional factors were also examined (tertiles): total daily energy intake, protein, carbohydrate, starch, sugar, fibers, lipid, fatty acids (total, *trans*, saturated, monounsaturated, polyunsaturated) intakes (as residuals on total energy intake), glycemic index (GI) and glycemic load (GL), dietary patterns (“Fruits and Vegetables,” “Western,” “Modern Mexican”), and the HEI score. Heterogeneity of means across categories was assessed by F test from analyses of variance for all 46 variables, and *P* values were corrected for multiple tests with a Bonferroni correction (*P* < 0.001(0.05/46)). When significant heterogeneity was detected, linear trend across ordinal categories was further tested by assigning the median value of each category to participants and including the variable as a continuous term in a linear regression model.

All statistical tests were two-sided. Analyses were performed using SAS 9.4 (SAS Institute, Cary, NC) and R Studio (packages *NPC* [35] and *glmnet* [36]).

## Results

As shown in Table 1, mean percent mammographic density was 36.5 (SD = 17.0) % and mean age at mammography was 43.1 (SD = 3.7) years. Fifty-two percent of women had already used an oral contraceptive, and women had on average 2.1 (SD = 1.2) children, with a mean age at first full-term pregnancy of 24.8 (SD = 4.4) years. Only 10.4% of parous participants had never breastfed. Mean BMI was 28.4 (SD = 5.4) kg/m^2^ and 70.7% of participants were overweight or obese (BMI ≥ 25 kg/m^2^). Mean concentration of metabolites are shown in Supplementary Table 2.

**Table 1:** Selected characteristics of the population (*N* = 573)

Characteristics	Missing values (***N***)	Mean (SD) or ***N*** (%)
**State of recruitment**	0	
Jalisco		318 (55.5)
Veracruz		255 (44.5)
**Breast density (%)**	0	36.5 (17.0)
**Dense tissue area (cm**^**2**^**)**	0	48.4 (33.3)
**Non-dense area (cm**^**2**^**)**	0	82.3 (38.5)
**Age at mammography (years)**	0	43.1 (3.7)
**Age at menarche (years)**	4	12.6 (1.5)
**Family history of breast cancer (yes)**	0	27 (4.7)
**History of benign breast disease (yes)**	0	79 (13.8)
**Oral contraceptive use (ever)**	19	288 (52.0)
**Number of full-term pregnancies**	30	2.1 (1.2)
**Age at first birth**^**a**^ **(years)**	43	24.8 (4.4)
**Duration of breastfeeding**^**a**^	41	
No breastfeeding		49 (10.4)
< 6 months		96 (20.4)
6 to 12 months		100 (21.3)
12 to 24 months		133 (28.3)
≥ 24 months		92 (19.6)
**Physical activity (MET hours/week)**	1	26.5 (21.1)
**Alcohol intake (drinks/day)**	45	
0		160 (30.3)
< 0.1		259 (49.1)
0.1-0.2		76 (14.4)
≥ 0.2		33 (6.3)
**Smoking status**	62	
Never		366 (71.6)
Past		87 (17.0)
Current		58 (11.4)
**BMI (kg/m**^**2**^**)**	0	28.4 (5.4)
< 25		168 (29.3)
25-30		214 (37.4)
≥30		191 (33.3)
**Socioeconomic status**	79	
Low		84 (17.0)
Medium		216 (43.7)
High		194 (39.3)

^a^Among parous women (*n* = 511)

Abbreviations: *BMI* body mass index; *SD* standard deviation

Pearson’s correlation coefficients between percent mammographic density and dense area, non-dense area, age and BMI were respectively 0.75, − 0.54, − 0.13, and − 0.16 (all *P* values< 0.002, not shown). Moderate positive correlations were observed among amino acids, acylcarnitines, and phosphatidylcholines (with respective average correlations of 0.40, 0.31, and 0.37, not tabulated) and were stronger among lysophosphatidylcholines and sphingomyelins (with respective average correlations of 0.57 and 0.67, not tabulated) (Supplementary Figure 1).

### Metabolites associated with mammographic density

**Fig. 1:**
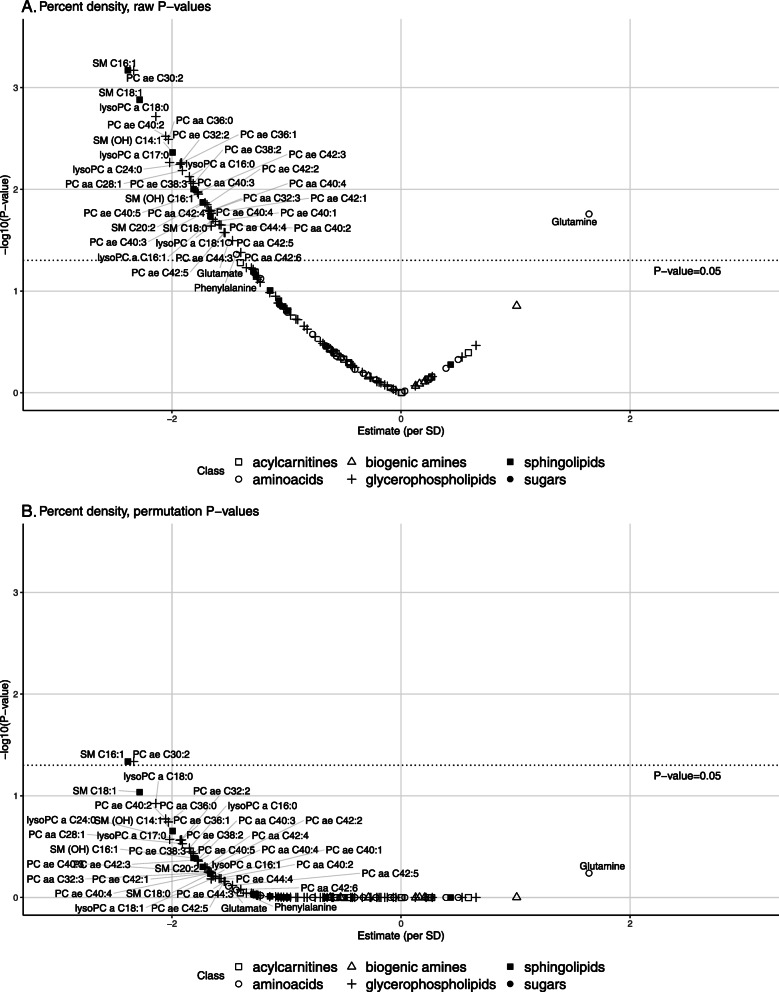
Associations between metabolites and percent mammographic density (PMD). **A** Raw *P* values. **B** Adjusted *P* values. Estimates per standard deviation increase in residuals of metabolites on batch were obtained from linear regression adjusted for age, BMI, age at menarche, family history of cancer, history of benign breast disease, use of oral contraceptive, number of full-term pregnancies, age at first full-term pregnancy, breastfeeding, alcohol intake, smoking status, socioeconomic status, and physical activity. Dotted lines represent statistical significance thresholds for raw *P* values (**A**) and for *P* values adjusted by permutation-based stepdown *minP* (**B**). ae, acyl-alkyl; aa, acyl-acyl; PC, phosphatidylcholine; SM, sphingomyelin; SD, standard deviation

Associations of metabolites and percent mammographic density before correction for multiple tests are shown in Figure 1A. After correction of *P* values for multiple tests, only two inverse associations remained borderline statistically significant, SM C16:1 (*minP P* value = 0.05) and PC ae C30:2 (*minP P* value = 0.05) (Figure 1B). *P* values for tests of linear trends across quartiles of metabolites were < 0.01 for both SM C16:1 and PC ae C30:2 (Table 2). For SM C16:1, mean percent mammographic density was 38.1 (95% confidence interval (CI) 32.2-44.0) in first quartile and 32.0 (26.3–37.8) in last quartile. For PC ae C30:2, mean percent mammographic density was 40.7 (34.9–46.5) in the first quartile and 35.0 (29.2–40.8) in last quartile.

**Table 2:** Adjusted means of PMD across quartiles of PCaeC30:2 and SMC16:1, overall and by BMI

		Quartiles of metabolites plasma concentrations				
		Q1	Q2	Q3	Q4	
Metabolite		Adjusted mean PMD^1^ (95 % CI)	Adjusted mean PMD^1^ (95% CI)	Adjusted mean PMD^1^ (95% CI)	Adjusted mean PMD^1^ (95% CI)	*P trend*^*2*^
SM C16:1	Overall	38.1 (32.2–44.0)	39.8 (34.1–45.5)	36.6 (30.9–42.2)	32.0 (26.3–37.8)	*< 0.01*
	BMI (kg/m^2^)					
*n* = 286	< 27.4	43.7 (35.7–51.8)	41.7 (33.7–49.7)	39.6 (31.8–47.3)	35.1 (27.1–43.1)	*< 0.01*
*n* = 287	≥ 27.4	29.6 (19.1–40.1)	35.4 (25.3–45.6)	29.7 (19.8–39.5)	25.9 (15.7–36.1)	*0.07*
					*P* interaction^3^	0.51
PC ae C30:2	Overall	40.7 (34.9–46.5)	37.6 (31.8–43.5)	34.5 (28.8–40.1)	35.0 (29.2–40.8)	*< 0.01*
	BMI (kg/m^2^)					
*n* = 286	< 27.4	42.4 (34.4–50.4)	42.2 (34.1–50.2)	36.8 (29.0–44.6)	38.5 (30.4–46.7)	*0.09*
*n* = 287	≥ 27.4	36.8 (26.2–47.3)	31.6 (21.4–41.9)	29.8 (20.0–39.6)	28.6 (18.3–38.8)	*< 0.01*
					*P* interaction^3^	0.31

Abbreviations: *ae* acyl-alkyl; *BMI* body mass index; *CI* confidence interval; *PC* phosphatidylcholine; *PMD* percent mammographic density; *Q* quartile; *SM* sphingomyelin

^1^Means were adjusted for age, BMI, age at menarche, family history of cancer, history of benign breast disease, use of oral contraceptive, number of full-term pregnancies, age at first full-term pregnancy, breastfeeding, alcohol intake, smoking status, socioeconomic status, and physical activity

^2^*P* value for test of linear trend across quartiles of metabolite, performed by assigning participants the median value in each quartile and modeling the corresponding variable as a continuous term

^3^*P* value for interaction term between residuals of log-transformed metabolites concentrations regressed on batch and dichotomized BMI (< 27.44/≥ 27.44)

None of the metabolites were associated with dense area after correction for multiple tests (data not shown, all *minP P* values > 0.69).

For non-dense area, after correction for multiple tests, 16 metabolites remained associated with non-dense area: 12 PCs (ae C30:2, ae C40:2, ae C40:5, ae C32:2, aa C36:0, aa C28:1, ae C42:2, ae C42:5, ae C38:3, ae C40:4, aa C40:3, aa C42:4) and 4 SMs (C18:1, C16:1, C20:2, OH-C14:1) (Supplementary Figure 2), with *P* values for tests of linear trends across quartiles all ≤0.02 (data not shown).

When assessing the associations of percent mammographic density with SM C16:1 and PC ae C30:2 across BMI strata (Table 2), no statistically significant interaction was detected for any of the metabolites (*P* interaction≥0.31). Of note, a linear inverse association with percent mammographic density was observed among quartiles of SM C16:1 (*P* trend< 0.01) in women with a BMI lower than 27.4 kg/m^2^ (*n* = 286), but not in women with a BMI ≥ 27.4 kg/m^2^ (*n* = 287, *P* trend = 0.07). For PC ae C30:2, no statistically significant linear trend across quartiles was observed among women with a BMI < 27.4 kg/m^2^ (*P* trend = 0.09), while a significant inverse association was observed among women with a BMI ≥ 27.4 kg/m^2^ (*P* trend = < 0.01). There was no evidence of a statistical interaction between any of the other metabolites and BMI (dichotomized by median) (all *P* interaction ≥0.07) for percent mammographic density, and none of the other metabolites was associated with percent mammographic density in any of the BMI strata after adjustment for multiple tests (data not shown).

### Exploratory analyses

In exploratory analyses using cross-validated bootstrapped LASSO, SM C16:1 was the most frequently (in 84% of bootstrap samples) identified metabolite. In contrast, PC ae C30:2 was selected in the signature in only 18% of bootstrap samples.

### Correlates of metabolites associated with percent mammographic density PMD

**Fig. 2:**
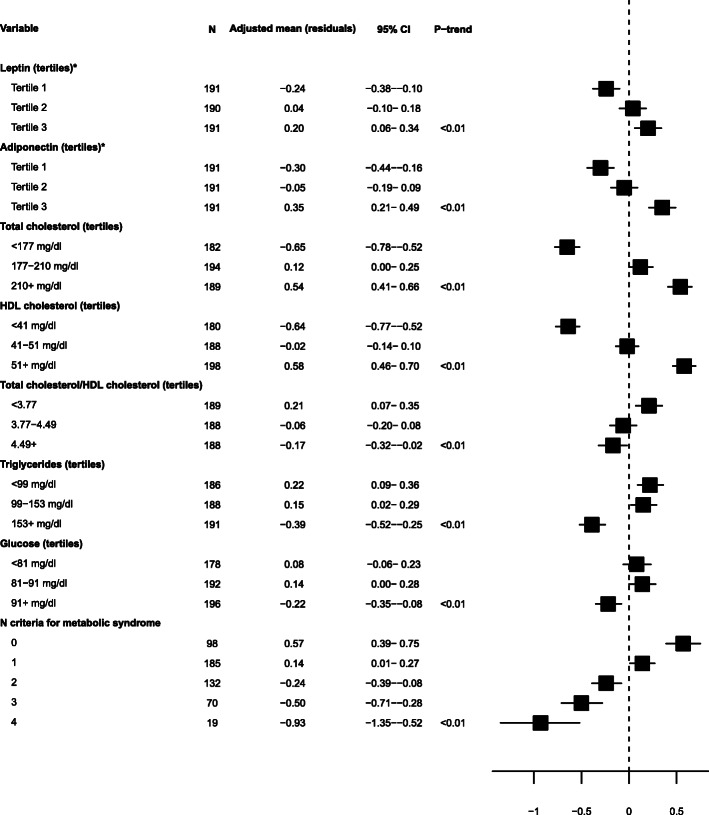
Adjusted mean concentrations of SMC16:1 by levels of lifestyle, anthropometric, metabolic, and dietary factors. Adjusted means of SM C16:1 (residuals of log-transformed concentration regressed on analytical batch) were obtained from models adjusted for age and state. Variables shown here are the ones for which a significant heterogeneity was detected by F test from analysis of variance, based on Bonferroni-corrected *P* values. Linear trend across ordinal categories was tested by assigning the median value of each category to participants and including the variable as a continuous term in a linear regression model. Asterisk indicates tertiles of residuals of leptin and adiponectin concentrations regressed on analytical batches. CI, confidence interval; HDL, high-density lipoprotein; SM, sphingomyelin

**Fig. 3:**
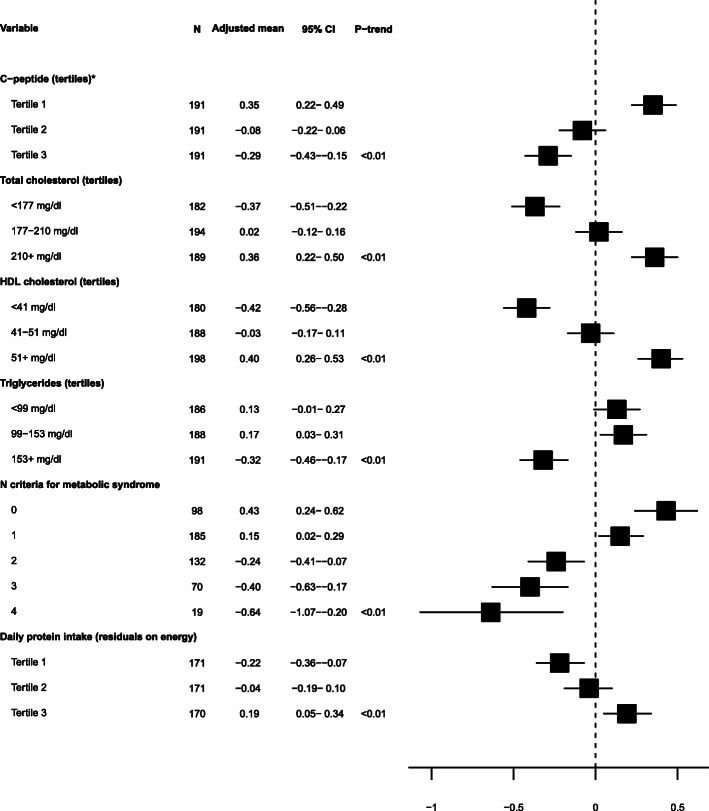
Adjusted mean concentrations of PC ae C30:2 by levels of lifestyle, anthropometric, metabolic, and dietary factors. Adjusted means of PC ae C30:2 (residuals of log-transformed concentration regressed on analytical batch) were obtained from models adjusted for age and state. Variables shown here are the ones for which a significant heterogeneity was detected by F test from analysis of variance, based on Bonferroni-corrected *P* values. Linear trend across ordinal categories was tested by assigning the median value of each category to participants and including the variable as a continuous term in a linear regression model.. Asterisk indicates tertiles of residuals of C-peptide concentrations regressed on analytical batches. CI, confidence interval; HDL, high-density lipoprotein; PC ae, phosphatidylcholine acyl-alkyl

Of all potential correlates investigated, only those for which a statistically significant association was detected after correction of *P* values (from F-tests) for multiple tests are shown in Figures 2 and 3.

Figure 2 shows adjusted mean concentrations of SM C16:1 (residuals) across categories of variables for which a statistically significant association was detected after correction of *P* values (from F-tests) for multiple tests and shows *P* for linear trend (all < 0.01) for these variables: leptin, adiponectin, total cholesterol, HDL cholesterol (direct associations), total/HDL cholesterol ratio, triglycerides, glucose, and number of components for metabolic syndrome (inverse associations).

For PC ae C30:2 (Figure 3), significant heterogeneity after correction for multiple tests of *P* values from F-tests was observed across categories of C-peptide, triglycerides, number of components for metabolic syndrome (inverse associations), total cholesterol, HDL cholesterol, and daily protein intake (direct associations).

No statistical interaction was observed with state of residence for the two metabolites. Factors associated with SM C16:1 in the whole population remained associated at a statistically significant level in stratified analyses, except for leptin and total cholesterol/HDL cholesterol ratio, for which the associations were no longer significant after Bonferroni correction in both states, and glucose, which was no longer significant in Jalisco after Bonferroni correction. Total cholesterol, HDL cholesterol, and number of components of metabolic syndrome remained associated with PC ae C30:2 after Bonferroni correction in each state, while the association with C-peptide and triglycerides were significant in Jalisco only, and protein intake was not associated with PC ae C30:2 in any of the states.

## Discussion

In this population of premenopausal Mexican women, lower plasma concentrations of sphingomyelin C16:1 and phosphatidylcholine acyl-alkyl C30:2 were associated with a higher percent mammographic density. Analyses of a wide range of lifestyle, dietary, anthropometric, and metabolic factors indicated associations of these two metabolites with mostly metabolic parameters.

Indeed, for SM C16:1, direct associations were observed with total and HDL cholesterol, leptin, and adiponectin and inverse associations with total cholesterol/HDL cholesterol ratio, triglycerides, glucose, and number of criteria for metabolic syndrome. For PC ae C30:2, direct associations with total and HDL cholesterol were observed, as well as with dietary protein intake, and inverse associations were observed with C-peptide, triglycerides, and number of criteria for metabolic syndrome.

We could not identify previous publications reporting the associations of SM C16:1 and PC ae C30:2 with mammographic density, nor any studies applying metabolomics to study mammographic density.

Sphingomyelins are abundant in lipoproteins and have a key role in the transport of cholesterol, especially in very low-density lipoproteins (VLDL) [37]. HDL also contributes to plasma concentrations of SM [37, 38]. Some SMs and their precursors, ceramides, have been associated with cardiovascular disease risk, type II diabetes, and obesity [37, 39, 40]. SMs have been associated with ovarian cancer risk [41], and some SMs (but not SM C16:1) were inversely associated with breast cancer risk before correction of *P* values for multiple tests, in a metabolomics study including pre- and postmenopausal women [17]. A Mendelian randomization (MR) study of SMs and breast cancer risk reported a null association with breast cancer [42], while an MR study of SMs in breast cancer survival indicated an inverse association with risk of breast cancer-specific death (in women with estrogen-receptor positive tumors) [43]. Sphingolipids are involved in cancer cell death signaling [44], in particular through regulatory actions of SMs and their ceramide precursors on apoptosis [45, 46]. Interestingly, in the latter MR study, a sensitivity analysis, based on detected pleiotropic associations of genetic instruments for SMs with cholesterol, highlighted that single nucleotide polymorphisms associated with cholesterol, in particular LDL cholesterol, were strongly associated with circulating SMs. These findings are in line with our analysis on correlates of SM C16:1 showing strong associations with total and HDL cholesterol, and inverse associations with the ratio of total and HDL cholesterol. These associations are consistent with previous work in this population [15, 23]. Other factors associated with SM C16:1 were circulating leptin and adiponectin concentrations (but not their ratio). These two adipokines, not correlated with each other in our population [15], are involved in metabolic health and could influence ceramide levels [47].

Studies have reported lower levels of acyl-alkyl phosphatidylcholines in diabetic patients compared to non-diabetic individuals (although the observed associations were not specific to PC ae C30:2) [48–50], which is consistent with the inverse association observed with C-peptide in our analysis. While hepatic diacyl-PCs play a role in regulating the efflux of lipoprotein secretion, in particular VLDL, from the liver [51], acyl-alkyl PCs may prevent oxidation of lipoproteins [52]. PCs are synthesized from choline [53], an essential nutrient whose main sources, as estimated from the US National Health and Nutrition Examination Survey [54], are protein-rich foods. This is consistent with the direct association we observed with dietary protein intake. Acyl-alkyl PCs have been inversely associated with risk of breast cancer in a prospective study [17]. However, the biological mechanism underpinning the association of this metabolite with mammographic density remains unclear. In our analysis, both SM C16:1 and PC ae C30:2 concentrations decreased with the number of criteria for the metabolic syndrome, which could partly result from the associations described above with various metabolic parameters, including total and HDL cholesterol. Of note, none of the anthropometric factors investigated were associated with these two metabolites after multiple test correction.

In this analysis, we were able to investigate the associations between several circulating metabolites and mammographic density measured at the same time, accounting for BMI and other potential confounders for which data have been collected in the Mexican Teachers’ Cohort. The study design also allowed us to explore potential lifestyle, dietary, anthropometric, and metabolic correlates of key metabolites. In addition, women included in this study were not using oral contraceptives at the time of clinical examination, which is a strength given previous reports of different associations between metabolites and breast cancer risk according to hormone use [17], and blood was collected in fasting state for all women. Nevertheless, an important limitation of this work is that blood samples were collected only once, hence raising reproducibility issues regarding metabolites concentrations. However, studies have shown the metabolite concentrations to be relatively stable over 4 months to 2 years for most metabolites in this work [55–57]. Another limitation regarding metabolites measurements arises from the method used for some class of compounds. Indeed, the signal observed is not specific and may correspond to several compounds, which we are not able to distinguish. Therefore, additional studies with more specific methods are required.

## Conclusions

In conclusion, our work showed that two plasma metabolites, SM C16:1 and PC ae C30:2, were inversely associated with percent mammographic density among premenopausal Mexican women. These metabolites are both correlated with several biomarkers of metabolic health, which may provide new perspectives to support future preventive actions for breast cancer. Further work is needed to evaluate whether these two metabolites can bring useful information for the identification of women with dense breasts.

## Supplementary Information

**Additional file 1: **Supplementary tables describing the completeness of the metabolites measures and coefficients of variations (**Supplementary Table 1**) and geometric mean of metabolites concentrations (**Supplementary Table 2**); Supplementary figures showing correlations between metabolites adjusted for age, batch, and state (**Supplementary Figure 1**) and associations between metabolites and non-dense area (**Supplementary Figure 2**).

## Data Availability

Access to the Mexican Teachers’ Cohort resources can be requested using the dedicated form available at http://www.esmaestras.org/investigadores/acceso_propuestas.php. Eligible individuals must be affiliated to an academic or research institution. Outside users are advised to informally consult MTC’s principal investigator Dr. Lajous [mlajous@insp.mx] regarding the availability of data to address a specific research question. After preliminary approval, outside users prepare and submit a brief proposal that is reviewed by the MTC scientific committee.

## Abbreviations

BMI: Body mass index
CI: Confidence interval
CRP: C-reactive protein
GI: Glycemic index
GL: Glycemic load
HDL: High density lipoprotein
HEI: Healthy eating index
IARC: International agency for research on cancer
IGF-1: Insulin-like growth factor 1
IGF-BP3: Insulin-like growth factor binding protein 3
LLOQ: Lower limit of quantification
LOD: Limit of detection
PC: Phosphatidylcholine
SM: Sphingomyelin;
ULOQ: Upper limit of quantification
