# Supplementary material for: Biomarkers of mammographic density in premenopausal women
Source: Breast Cancer Res. 2021 Jul 23;23:75. doi: 10.1186/s13058-021-01454-3 (PMC8305592; doi:10.1186/s13058-021-01454-3)

Supplementary Table 1. Completeness and exclusions of the different metabolites, with limits of detection (LOD) and limits of quantification (LOQ)

Metabolites[1]	Results outside the measurable range (N=599 samples)		CV, % [3]
	Samples, %	Criteria for results considered out of the measurable range[2]	
**Acylcarnitines**			
C0	0.0	<LOD	3.5
C2	0.0	<LOD	3.9
C3	0.0	<LOD	4.3
C3-DC (C4-OH)	41.5	<LOD	-
C4	0.0	<LOD	4.7
C5	9.0	<LOD	6.2
C10	62.7	<LOD	-
C10:1	38.8	<LOD	-
C12	37.8	<LOD	-
C12:1	0.2	<LOD	8.5
C14:1	0.0	<LOD	5.0
C14:2	7.5	<LOD	5.1
C16	0.0	<LOD	4.3
C16:1	77.5	<LOD	-
C18	0.0	<LOD	7.0
C18:1	0.0	<LOD	4.8
C18:2	0.0	<LOD	5.9
**Amino acids**			
Alanine	0.0	<LLOQ (< 20 µmol/L)	4.0
Arginine	1.8	>ULOQ (> 400 µmol/L)	4.4
Asparagine	0.0	<LLOQ (< 5 µmol/L)	4.9
Aspartate	0.0	<LLOQ (< 5 µmol/L)	11.5
Citrulline	0.0	<LLOQ (< 5 µmol/L)	5.2
Glutamine	0.0	<LLOQ (< 20 µmol/L)	5.3
Glutamate	0.0	<LLOQ (< 10 µmol/L)	6.0
Glycine	0.0	<LLOQ (< 25 µmol/L)	7.4
Histidine	0.0	<LLOQ (< 5 µmol/L)	3.9
Isoleucine	0.0	<LLOQ (< 5 µmol/L)	6.2
Leucine	0.0	<LLOQ (< 50 µmol/L)	4.9
Lysine	0.0	<LLOQ (< 10 µmol/L)	7.5
Methionine	0.0	<LLOQ (< 5 µmol/L)	9.6
Ornithine	0.0	<LLOQ (< 5 µmol/L)	7.5
Phenylalanine	0.0	<LLOQ (< 5 µmol/L)	4.1
Proline	0.0	<LLOQ (< 10 µmol/L)	4.7
Serine	0.0	<LLOQ (< 5 µmol/L)	4.0
Threonine	0.0	<LLOQ (< 5 µmol/L)	4.0
Tryptophane	0.0	<LLOQ (< 5 µmol/L)	4.3
Tyrosine	0.0	<LLOQ (< 5 µmol/L)	3.8
Valine	0.0	<LLOQ (< 10 µmol/L)	9.1
**Biogenic amines**			

ADMA	0.0	<LLOQ (< 0.25 µmol/L)	10.4
Creatinine	0.0	<LLOQ (< 10 µmol/L)	3.7
Kynurenine	0.5	<LLOQ (< 1 µmol/L)	7.5
Putrescine	73.8	<LLOQ (< 0.1 µmol/L)	-
SDMA	0.0	<LLOQ (< 0.1 µmol/L)	7.3
Sarcosine	48.5	<LLOQ (< 1 µmol/L)	-
Serotonin[4]	1.0	<LLOQ (< 0.1 µmol/L)	-
Spermidine	54.2	<LLOQ (< 0.25 µmol/L	-
Spermine	94.5	<LLOQ (< 0.25 µmol/L	-
Taurine	17.3	>ULOQ (> 200 µmol/L)	3.4
alpha-AAA	77.8	<LLOQ (< 1 µmol/L)	-
t4-OH-Pro	0.0		5.8
**Glycerophospholipids**			
PC aa C28:1	0.0	<LOD	6.1
PC aa C30:0	0.0	<LOD	6.2
PC aa C32:0	0.0	<LOD	6.1
PC aa C32:1	0.0	<LOD	5.9
PC aa C32:2	0.0	<LOD	5.5
PC aa C32:3	0.0	<LOD	6.1
PC aa C34:1	0.0	<LOD	4.6
PC aa C34:2	0.0	<LOD	4.6
PC aa C34:3	0.0	<LOD	5.3
PC aa C34:4	0.0	<LOD	5.1
PC aa C36:0	0.0	<LOD	6.3
PC aa C36:1	0.0	<LOD	5.7
PC aa C36:2	0.0	<LOD	4.6
PC aa C36:3	0.0	<LOD	5.3
PC aa C36:4	0.0	<LOD	4.7
PC aa C36:5	0.0	<LOD	4.8
PC aa C36:6	0.0	<LOD	5.6
PC aa C38:0	0.0	<LOD	7.2
PC aa C38:3	0.0	<LOD	5.3
PC aa C38:4	0.0	<LOD	4.7
PC aa C38:5	0.0	<LOD	4.9
PC aa C38:6	0.0	<LOD	4.9
PC aa C40:1	39.8	<LOD	-
PC aa C40:2	0.0	<LOD	8.2
PC aa C40:3	0.0	<LOD	8.7
PC aa C40:4	0.0	<LOD	5.0
PC aa C40:5	0.0	<LOD	5.3
PC aa C40:6	0.0	<LOD	5.7
PC aa C42:0	0.0	<LOD	7.4
PC aa C42:1	0.0	<LOD	10.9
PC aa C42:2	0.0	<LOD	9.5
PC aa C42:4	0.0	<LOD	13.3
PC aa C42:5	0.0	<LOD	9.0

PC aa C42:6	0.0	<LOD	6.3
PC ae C30:0	0.0	<LOD	8.5
PC ae C30:2	0.0	<LOD	11.7
PC ae C32:1	0.0	<LOD	6.2
PC ae C32:2	0.0	<LOD	6.7
PC ae C34:0	0.0	<LOD	6.8
PC ae C34:1	0.0	<LOD	5.5
PC ae C34:2	0.0	<LOD	6.2
PC ae C34:3	0.0	<LOD	5.6
PC ae C36:0	3.2	<LOD	7.6
PC ae C36:1	0.0	<LOD	7.0
PC ae C36:2	0.0	<LOD	5.2
PC ae C36:3	0.0	<LOD	4.8
PC ae C36:4	0.0	<LOD	5.5
PC ae C36:5	0.0	<LOD	5.4
PC ae C38:0	0.0	<LOD	7.6
PC ae C38:2	0.0	<LOD	7.6
PC ae C38:3	0.0	<LOD	7.1
PC ae C38:4	0.0	<LOD	5.1
PC ae C38:5	0.0	<LOD	5.5
PC ae C38:6	0.0	<LOD	4.9
PC ae C40:1	0.0	<LOD	8.3
PC ae C40:2	0.0	<LOD	5.7
PC ae C40:3	0.0	<LOD	5.6
PC ae C40:4	0.0	<LOD	7.4
PC ae C40:5	0.0	<LOD	6.7
PC ae C40:6	0.0	<LOD	5.8
PC ae C42:1	0.0	<LOD	10.1
PC ae C42:2	0.0	<LOD	6.4
PC ae C42:3	0.0	<LOD	7.4
PC ae C42:4	0.5	<LOD	7.8
PC ae C42:5	0.0	<LOD	5.2
PC ae C44:3	0.0	<LOD	11.8
PC ae C44:4	0.0	<LOD	7.1
PC ae C44:5	0.0	<LOD	6.5
PC ae C44:6	0.0	<LOD	5.9
lysoPC a C16:0	0.0	<LOD	5.7
lysoPC a C16:1	0.0	<LOD	6.4
lysoPC a C17:0	0.0	<LOD	6.3
lysoPC a C18:0	0.0	<LOD	5.8
lysoPC a C18:1	0.0	<LOD	6.1
lysoPC a C18:2	0.0	<LOD	6.8
lysoPC a C20:3	0.0	<LOD	5.2
lysoPC a C20:4	0.0	<LOD	5.9
lysoPC a C24:0	0.0	<LOD	9.9
**Sphingolipids**			

SM C16:0	0.0	<LOD	6.1
SM C16:1	0.0	<LOD	6.3
SM C18:0	0.0	<LOD	7.2
SM C18:1	0.0	<LOD	6.4
SM C20:2	0.0	<LOD	9.7
SM C24:0	0.0	<LOD	6.0
SM C24:1	0.0	<LOD	6.5
SM C26:0	9.0	<LOD	11.7
SM C26:1	1.0	<LOD	10.7
SM (OH) C14:1	0.0	<LOD	6.5
SM (OH) C16:1	0.0	<LOD	6.2
SM (OH) C22:1	0.0	<LOD	7.0
SM (OH) C22:2	0.0	<LOD	5.8
SM (OH) C24:1	0.0	<LOD	9.9
**Hexose**			
Hexose	0.0	<LLOQ (< 1500 µmol/L)	3.4

[1]Metabolites in gray were excluded from the statistical analysis because more than 20% or the measures were outside the measurable range.
[2]Limit of detection (LOD) is applicable to acylcarnitines, glycerophospholipids, sphingolipids. Lower limit of quantification (LLOQ) or upper limit of quantification (ULOQ) is applicable to amino acids, biogenic amines and hexose. No metabolites had values both under LLOQ and over ULOQ.
[3]Mean intra- and inter-batch coefficients of variation (CVs) based on two quality control samples measured in duplicate in each batch.
[4]CV for serotonin not calculated because measures in quality controls were lower than LLOQ.

Supplementary Table 2. Geometric means of metabolites concentrations

**Variable**	N	**Geometric mean (95% CI) (µmol/L)**
**C0**	573	32.18 (31.69-32.69)
**C2**	573	5.31 (5.19-5.44)
**C3**	573	0.30 (0.29-0.31)
**C4**	573	0.15 (0.14-0.15)
**C5**	573	0.11 (0.11-0.12)
**C12:1**	573	0.21 (0.21-0.21)
**C14:1**	573	0.06 (0.06-0.06)
**C14:2**	573	0.03 (0.03-0.03)
**C16**	573	0.11 (0.11-0.11)
**C18**	573	0.05 (0.04-0.05)
**C18:1**	573	0.12 (0.12-0.13)
**C18:2**	573	0.06 (0.06-0.06)
**Alanine**	573	469.21 (461.08-477.48)
**Arginine**	573	157.23 (153.14-161.43)
**Asparagine**	573	51.05 (50.19-51.92)
**Aspartate**	573	51.50 (50.28-52.75)
**Citrulline**	573	25.86 (25.32-26.41)
**Glutamine**	573	491.29 (483.10-499.62)
**Glutamate**	573	153.86 (148.98-158.90)
**Glycine**	573	348.92 (341.28-356.73)
**Histidine**	573	99.11 (97.83-100.41)
**Isoleucine**	573	76.22 (74.96-77.51)
**Leucine**	573	155.26 (152.62-157.94)
**Lysine**	573	247.69 (243.53-251.93)
**Methionine**	573	23.47 (22.99-23.95)
**Ornithine**	573	93.29 (90.95-95.68)
**Phenylalanine**	573	99.09 (97.54-100.66)
**Proline**	573	201.05 (196.96-205.23)
**Serine**	573	179.38 (176.39-182.41)
**Threonine**	573	133.40 (130.66-136.19)
**Tryptophane**	573	63.24 (62.40-64.09)
**Tyrosine**	573	73.30 (72.02-74.61)
**Valine**	573	231.29 (227.26-235.39)
**ADMA**	573	0.47 (0.47-0.48)
**Creatinine**	573	53.09 (52.37-53.83)
**Kynurenine**	573	2.08 (2.04-2.12)
**SDMA**	573	0.42 (0.41-0.42)
**Serotonin**	573	0.67 (0.64-0.70)
**Taurine**	573	147.71 (144.49-151.00)
**t4-OH-Pro**	573	10.59 (10.18-11.01)
**PC aa C28:1**	573	1.78 (1.73-1.82)
**PC aa C30:0**	573	2.31 (2.25-2.37)
**PC aa C32:0**	573	17.31 (16.97-17.65)

**PC aa C32:1**	573	18.59 (17.95-19.24)
**PC aa C32:2**	573	6.75 (6.58-6.94)
**PC aa C32:3**	573	0.99 (0.97-1.01)
**PC aa C34:1**	573	238.47 (234.24-242.78)
**PC aa C34:2**	573	525.26 (518.64-531.96)
**PC aa C34:3**	573	25.11 (24.62-25.61)
**PC aa C34:4**	573	2.36 (2.29-2.42)
**PC aa C36:0**	573	2.64 (2.58-2.70)
**PC aa C36:1**	573	44.09 (43.19-45.00)
**PC aa C36:2**	573	288.26 (283.94-292.64)
**PC aa C36:3**	573	177.98 (175.08-180.92)
**PC aa C36:4**	573	197.37 (193.37-201.45)
**PC aa C36:5**	573	16.77 (16.21-17.34)
**PC aa C36:6**	573	0.94 (0.91-0.97)
**PC aa C38:0**	573	2.30 (2.25-2.35)
**PC aa C38:3**	573	49.27 (48.27-50.29)
**PC aa C38:4**	573	99.18 (97.06-101.34)
**PC aa C38:5**	573	48.30 (47.22-49.41)
**PC aa C38:6**	573	59.60 (58.12-61.12)
**PC aa C40:2**	573	0.48 (0.45-0.52)
**PC aa C40:3**	573	0.62 (0.59-0.65)
**PC aa C40:4**	573	3.43 (3.35-3.51)
**PC aa C40:5**	573	7.31 (7.14-7.48)
**PC aa C40:6**	573	18.15 (17.70-18.61)
**PC aa C42:0**	573	0.29 (0.28-0.29)
**PC aa C42:1**	573	0.16 (0.16-0.16)
**PC aa C42:2**	573	0.16 (0.15-0.17)
**PC aa C42:4**	573	0.18 (0.17-0.19)
**PC aa C42:5**	573	0.26 (0.25-0.26)
**PC aa C42:6**	573	0.28 (0.28-0.29)
**PC ae C30:0**	573	0.21 (0.20-0.21)
**PC ae C30:2**	573	0.08 (0.08-0.09)
**PC ae C32:1**	573	3.41 (3.34-3.49)
**PC ae C32:2**	573	1.04 (1.02-1.06)
**PC ae C34:0**	573	1.64 (1.60-1.68)
**PC ae C34:1**	573	12.64 (12.41-12.88)
**PC ae C34:2**	573	16.59 (16.24-16.94)
**PC ae C34:3**	573	10.13 (9.88-10.39)
**PC ae C36:0**	573	0.61 (0.58-0.63)
**PC ae C36:1**	573	14.13 (13.54-14.74)
**PC ae C36:2**	573	19.10 (18.72-19.49)
**PC ae C36:3**	573	10.47 (10.26-10.68)
**PC ae C36:4**	573	20.80 (20.34-21.28)
**PC ae C36:5**	573	11.87 (11.57-12.18)
**PC ae C38:0**	573	1.70 (1.66-1.73)
**PC ae C38:2**	573	4.49 (4.25-4.76)

**PC ae C38:3**	573	8.33 (7.93-8.75)
**PC ae C38:4**	573	14.14 (13.86-14.41)
**PC ae C38:5**	573	17.94 (17.56-18.32)
**PC ae C38:6**	573	6.55 (6.39-6.70)
**PC ae C40:1**	573	1.02 (0.99-1.05)
**PC ae C40:2**	573	1.77 (1.70-1.84)
**PC ae C40:3**	573	2.20 (2.05-2.36)
**PC ae C40:4**	573	3.12 (3.00-3.26)
**PC ae C40:5**	573	4.60 (4.43-4.77)
**PC ae C40:6**	573	3.54 (3.47-3.62)
**PC ae C42:1**	573	0.42 (0.40-0.43)
**PC ae C42:2**	573	0.42 (0.41-0.43)
**PC ae C42:3**	573	0.61 (0.59-0.63)
**PC ae C42:4**	573	0.74 (0.71-0.76)
**PC ae C42:5**	573	1.74 (1.69-1.79)
**PC ae C44:3**	573	0.09 (0.08-0.09)
**PC ae C44:4**	573	0.20 (0.19-0.20)
**PC ae C44:5**	573	0.68 (0.66-0.69)
**PC ae C44:6**	573	0.57 (0.56-0.59)
**lysoPC a C16:0**	573	156.23 (152.58-159.96)
**lysoPC a C16:1**	573	4.30 (4.20-4.41)
**lysoPC a C17:0**	573	2.96 (2.88-3.04)
**lysoPC a C18:0**	573	47.71 (46.49-48.96)
**lysoPC a C18:1**	573	25.45 (24.89-26.02)
**lysoPC a C18:2**	573	25.22 (24.68-25.77)
**lysoPC a C20:3**	573	2.08 (2.03-2.13)
**lysoPC a C20:4**	573	5.91 (5.75-6.07)
**lysoPC a C24:0**	573	0.11 (0.11-0.12)
**SM C16:0**	573	67.14 (65.77-68.54)
**SM C16:1**	573	10.49 (10.29-10.69)
**SM C18:0**	573	12.19 (11.92-12.46)
**SM C18:1**	573	6.78 (6.63-6.93)
**SM C20:2**	573	0.28 (0.27-0.28)
**SM C24:0**	573	6.60 (6.47-6.73)
**SM C24:1**	573	13.11 (12.82-13.40)
**SM C26:0**	573	0.03 (0.03-0.03)
**SM C26:1**	573	0.06 (0.06-0.07)
**SM (OH) C14:1**	573	3.84 (3.74-3.94)
**SM (OH) C16:1**	573	2.02 (1.97-2.07)
**SM (OH) C22:1**	573	5.16 (5.05-5.27)
**SM (OH) C22:2**	573	3.64 (3.56-3.73)
**SM (OH) C24:1**	573	0.39 (0.38-0.40)
**Hexose**	573	4423.7 (4334.1-4515.2)

Supplementary Figure 1. Pearson's partial correlations between metabolites adjusted for age, state, and batch

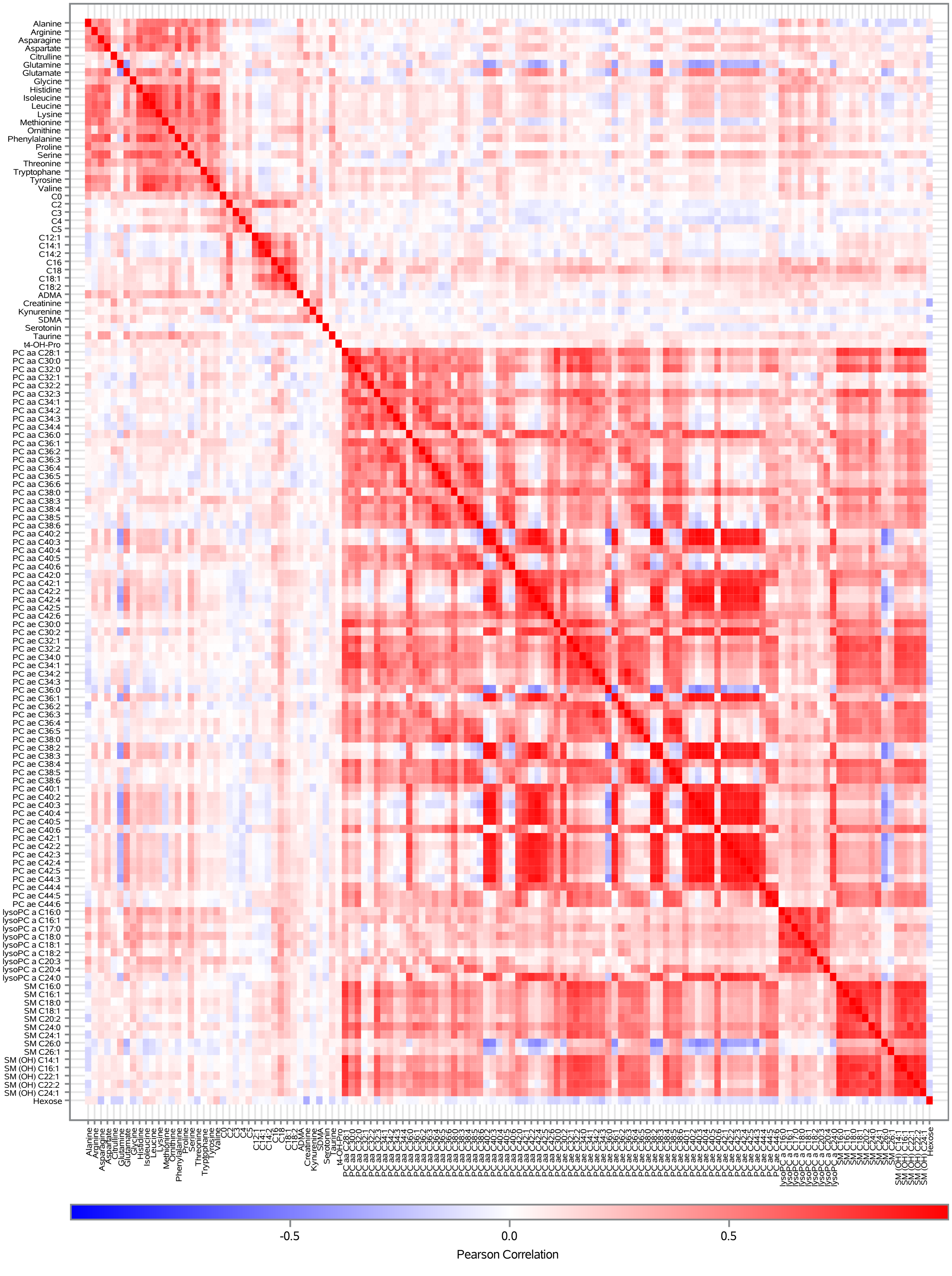

Supplementary Figure 2. Associations between metabolites and non-dense area. A: Raw P-values. B: Adjusted P-values. Estimates per standard deviation increase in residuals of metabolites on batch were obtained from linear regression adjusted for age, BMI, age at menarche, family history of cancer, history of benign breast disease, use of oral contraceptive, number of full-term pregnancies, age at first full-term pregnancy, breastfeeding, alcohol intake, smoking status, socioeconomic status, and physical activity. Dotted lines represent statistical significance thresholds for raw P-values (A) and for P-values adjusted by permutation-based stepdown *minP* (B). Abbreviations: ae: acyl-alkyl; aa: acyl-acyl; PC: phosphatidylcholine; SM: sphingomyelin; SD: standard deviation.

A.

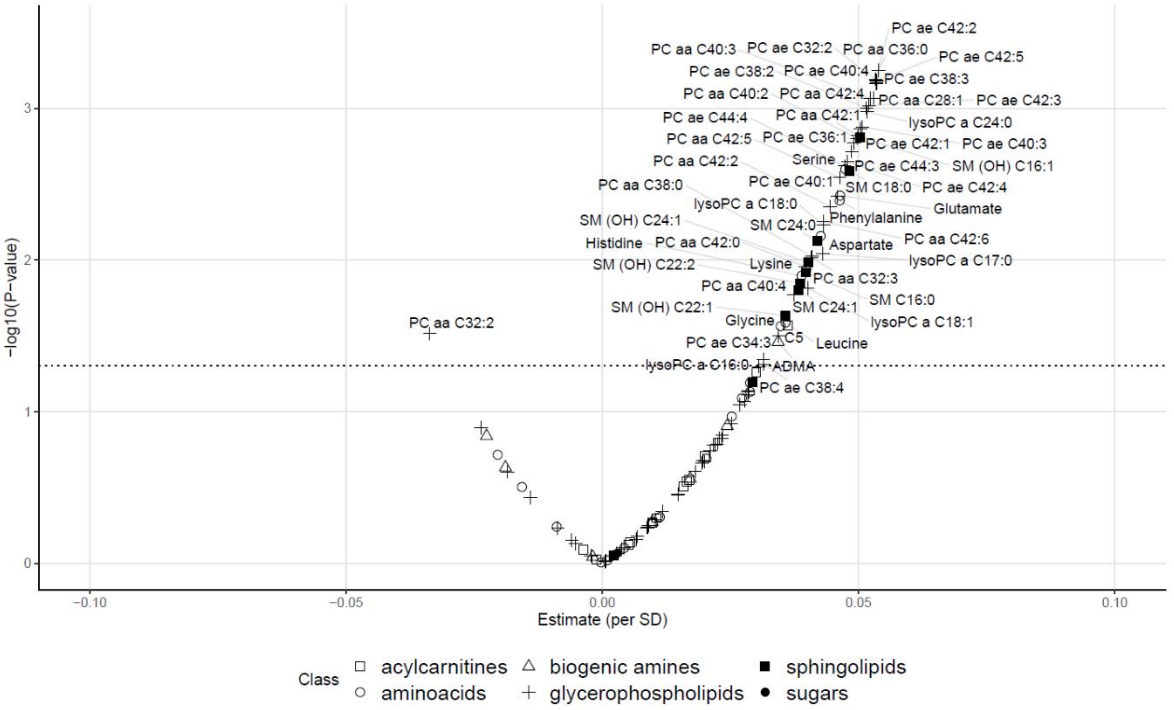

B.

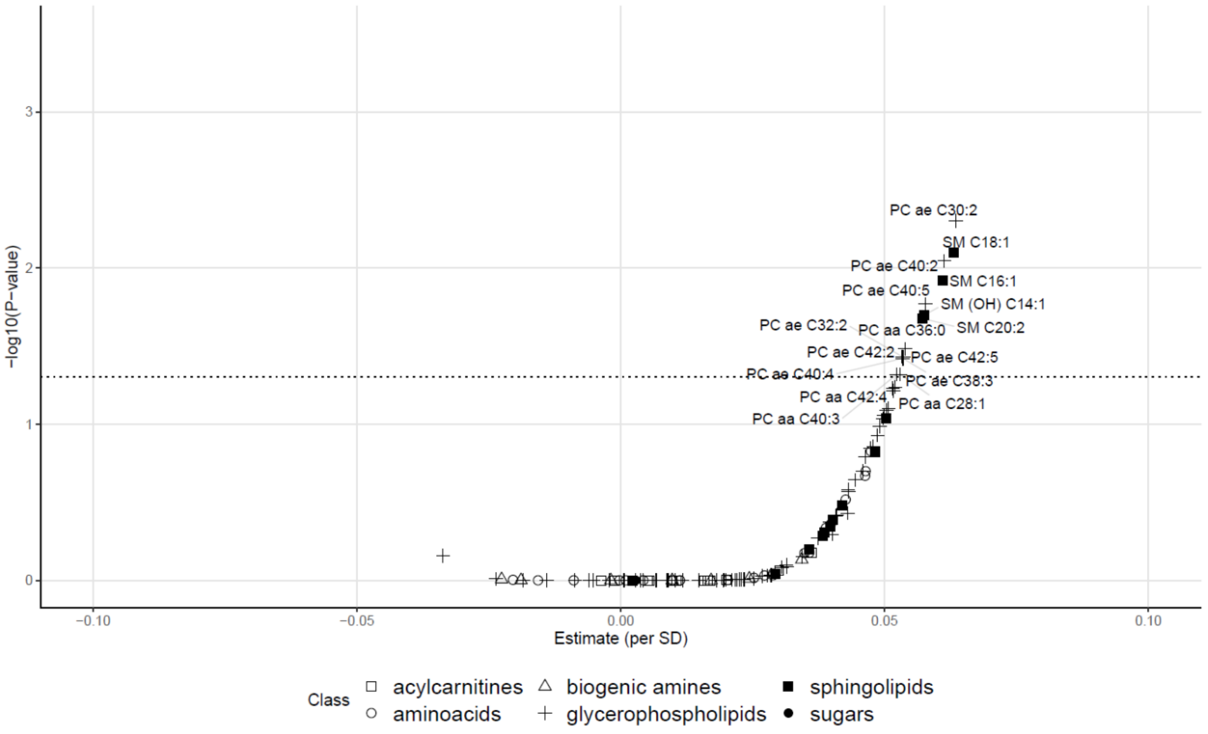

Supplement: Supplementary file 1 — Additional file 1: Supplementary tables describing the completeness of the metabolites measures and coefficients of variations (Supplementary Table 1) and geometric mean of metabolites concentrations (Supplementary Table 2); Supplementary figures showing correlations between metabolites adjusted for age, batch, and state (Supplementary Figure 1) and associations between metabolites and non-dense area (Supplementary Figure 2). [file 13058_2021_1454_MOESM1_ESM.pdf]
